# *Toxoplasma gondii* infection in HIV-infected pregnant women: epidemiology and risks of mother-to-child transmission

**DOI:** 10.11604/pamj.2022.42.275.33160

**Published:** 2022-08-12

**Authors:** Temi Lampejo

**Affiliations:** 1 Department of Infection Sciences, King’s College Hospital, London, United Kingdom

**Keywords:** *Toxoplasma gondii*, toxoplasmosis, HIV, pregnancy, congenital infection, reactivation

## Abstract

Toxoplasma gondii (T. gondii) infects approximately one third of the world’s population. Globally there are an estimated 13.1 million cases of T. gondii co-infection in HIV-infected people with 87.1% of these individuals living in sub-Saharan Africa. The risk of T. gondii infection in HIV-infected women rises significantly with lower CD4+ T cell counts (particularly under 100 cells/μl). Mother-to-child transmission (MTCT) occurs in approximately 30% of cases of maternal T. gondii infection during pregnancy. The global prevalence of latent toxoplasmosis in HIV-infected pregnant women is 47.5% but the overall risk in HIV-infected mothers of MTCT of T. gondii is however, estimated to be low at < 5%. MTCT in HIV-infected mothers not only occurs due to T. gondii primary infection in pregnancy but also due to reactivation. Infants with congenital toxoplasmosis born to HIV-infected mother may have a more rapid onset and greater dissemination of disease thus having potentially devastating effects. This article discusses the key risks for MTCT of T. gondii infection in HIV-infected mothers as well highlighting the many knowledge gaps for which further study is required.

## Commentary

The World Health Organisation (WHO) estimated that at the end of 2019 there were 38 million people living with HIV (81% of whom knew their HIV status) and that 68% of HIV-infected adults were receiving long-term antiretroviral therapy (ART) [1]. There were an estimated 1.3 million pregnant women living with HIV in 2018 of whom 1.1 million (84%) were receiving ART for prevention of mother-to-child transmission (MTCT) [2]. The obligate intracellular protozoan, *Toxoplasma gondii (T. gondii)*, infects approximately one third of the world´s population and has a worldwide distribution [3]. A recent systematic review estimated that globally there are an estimated 13.1 million cases of *T. gondii* co-infection in HIV-infected people with 87.1% of these individuals living in sub-Saharan Africa [4]. *T. gondii* acquisition is primarily through consumption of undercooked meat harbouring tissue cysts or contaminated food and water containing oocysts. Despite being preventable, *T. gondii* infection during pregnancy and subsequent congenital infection continues to devastate lives globally [5]. Acute toxoplasma infection during pregnancy can lead to abortion, intrauterine foetal loss and syndromes that include neurologic (including hydrocephaly/microcephaly) and neurocognitive deficits and chorioretinitis [6,7]. The risks of congenital infection due to *T. gondii* infection in HIV-infected pregnant women are amplified when compared to HIV-negative pregnant women potentially leading to even greater morbidity and mortality in this group of individuals, however, *T. gondii* infection in HIV-infected pregnant women remains a relatively neglected area of focus or study. This article therefore, aims to discuss the key risks for MTCT of *T. gondii* infection (and the potential underlying reasons for these risks) as well highlighting the many knowledge gaps in this subject area, which may give impetus for further studies and for the development of strategies to help mitigate the risks from this potentially devastating infection. This may also help HIV-infected pregnant mothers to receive appropriate and informed counselling regarding *T. gondii* infection in pregnancy.

The primary overall risk of MTCT of *T. gondii* is from acute *T. gondii* infection during pregnancy [8]. Maternal *T. gondii* infection greater than 3 months prior to conception confers minimal or no risk to the fetus. Transmission of *T. gondii* through breast milk has not been described in HIV-infected (or in HIV-uninfected) mothers. In pregnancy, maternal parasitaemia and subsequent invasion of the placenta by tachyzoites leads to the parasite entering the foetal circulation [9]. MTCT transmission occurs in approximately 30% of cases of maternal *T. gondii* infection during pregnancy [10]. Foetal infection rates are lower in early pregnancy (15% to 25% in the first trimester) but give rise to more severe congenital abnormalities whereas in the last trimester transmission rates are high (60% to 80%, up to 100% in the final few weeks) but typically does not result in congenital malformations although in some instances may cause isolated abnormalities (such as intracranial calcification or chorioretinitis) [10-12]. The timing of the *T. gondii* infection during pregnancy is therefore a major determinant of foetal outcome. A recent study found a global prevalence of acute *T. gondii* infection in pregnant women (including both HIV-positive and HIV-negative women) of 1.1% (0.6% when the most strict criteria for acute *T. gondii* infection were used); highest in the Eastern Mediterranean region (2.5%) and lowest in the European region (0.5%) [7]. They found that the prevalence of acute *T. gondii* infection was significantly higher in the first trimester (1.7%) compared to the second (1.0%) and third (0.1%) trimesters. The study did not report rates of MTCT/congenital toxoplasmosis or provide specific data regarding HIV-infected pregnant women. They did however estimate that based on an overall MTCT risk of approximately 24% in acute maternal *T. gondii* infection, annually 201,600 children could be born with congenital toxoplasmosis.

The risk of *T. gondii* infection in HIV-infected women rises substantially with CD4+ T lymphocyte counts of <100 cells/µl. The risk of MTCT of this parasite in HIV-infected mothers is not precisely known but is likely to be, at least to some extent, related to the degree of immunosuppression [13]. The overall risk in HIV-infected mothers of MTCT of *T. gondii* is however, estimated to be low at <5% [13]. MTCT risk in the setting acute maternal *T. gondii* infection is largely unknown for HIV-infected pregnant women. MTCT typically occurs due to a primary *T. gondii* infection occurring during pregnancy, however, in HIV-1 infected mothers, reactivation of latent *T. gondii* infection is also an important cause of congenital infection [10,13]. Moreover, this is not confined to mothers with low CD4 counts (<200 cells/mm^3^) [14]. At least eight cases of MTCT due to *T. gondii* reactivation in pregnancy in HIV-infected mothers have been reported in the literature [14-19] although several more instances are likely to have occurred. Toxoplasma serology serves as the mainstay of diagnosis of maternal *T. gondii* infection in pregnancy, however, in HIV-infected mothers who may be highly immunosuppressed, serological markers can be less reliable, further complicated by the fact that *T. gondii* IgM responses may be diminished in cases reactivation/reinfection when compared to primary infection. Three *T. gondii* lineages (type I, II and III) exist and re-infection with a different strain is another potential cause of congenital infection [10]. The majority of *T. gondii* strains isolated from HIV-infected individuals are type II [9]. Type I and type II strains have been identified in cases of congenital toxoplasmosis [9].

A recent meta-analysis estimating the prevalence of toxoplasmosis in HIV-infected pregnant women found an overall pooled prevalence of latent toxoplasmosis of 47.5% [20]. This study included a total of 3,256 HIV-infected pregnant women across 9 countries spanning Africa, South America and South-East Asia (with prevalence rates ranging from 8.7% in Zambia to 91.3% in Ethiopia). This is higher than the estimated global prevalence of latent toxoplasmosis of 33.8% in healthy pregnant women (known HIV-positive women were excluded from this meta-analysis) [7], and also higher than the estimated worldwide pooled toxoplasmosis prevalence of 35.8% in all HIV-infected individuals [4]. These findings are not entirely unexpected given that HIV-associated immunosuppression (through CD4 T-cell depletion, impaired cytotoxic T-lymphocyte activity and impaired IL-12 and INF-gamma production [21]) coupled with pregnancy-associated immunological changes may confer increased susceptibility to infection with *T. gondii* in this patient population. A Brazilian study of 2421 HIV-negative pregnant women and 168 HIV-positive pregnant women found that although high *T. gondii*-specific IgG values were seen more frequently in the HIV-positive pregnant women, this was not associated with an increased risk of MCTD of *T. gondii* [22].

There is a lack of studies comparing outcomes between infants congenitally infected with *T. gondii* that are born to HIV-infected mothers versus those born to HIV-negative mothers, however, infants with congenital toxoplasmosis born to HIV-infected mother appear to have a more rapid and disseminated disease process (including fever, failure to thrive, hepatosplenomegaly and seizures) [23]. There is contradictory and therefore inconclusive evidence regarding antenatal therapy such as pyrimethamine-sulfadiazine aimed at reducing the risk of MTCT of *T. gondii*, with some studies reporting a risk reduction but others showing no benefit [24]. The risk of MTCT transmission is thought to be reduced by up to approximately 60% with the use of spiramycin [25], however, spiramycin does not readily cross the placenta and therefore can only be used as prophylaxis in pregnancy prior to foetal transmission [19].

An additional consideration is the fact that for definitive confirmation of MTCT of *T. gondii* during pregnancy amniocentesis is required. In the pre-HAART era amniocentesis was discouraged in HIV-infected mothers due to the risks of MTCT transmission of HIV. A recent large multicentre case series by Florida *et al*. in which 88 HIV-infected pregnant underwent either amniocentesis (n = 79) or chorionic villous sampling (n = 9) found that no vertical transmission of HIV occurred in the 86 women that were on HAART [26]. Two cases of vertical transmission did occur in women not on HAART at the time of the invasive procedure [26]. Therefore, amniocentesis in HIV-infected mothers on HAART and virally suppressed are not deemed to be at a significantly increased risk of transmission of HIV to their foetus. It is not clear however, if in the context of co-infection with *T. gondii* this risk is altered. Placental invasion by *T. gondii* results in placental inflammation and whether this potentially renders the placental tissue more permissive to other pathogens such as HIV has not been determined. It has also been hypothesised that HIV-1 infected mothers, due to immune suppression, may in fact mount a diminished placental inflammatory response [27]. Mwanyumba *et al*. studied the effect of placental membrane inflammation due to acute chorioamnionitis in HIV-1 infected mothers in Kenya and found no association between chorioamnionitis and in utero transmission of HIV-1 although there was an association with peripartum HIV-1 transmission [28]. Extrapolating data from a study of another parasitic infection in HIV-1 infected pregnant women, histopathological analysis of placental tissue from 372 HIV-1 infected mothers infected with the parasite Plasmodium falciparum found no correlation between placental malaria and in utero (or peripartum) transmission of HIV-1 [29]. However, a retrospective study in Brazil on MTCD HIV transmission reported 7.46 times increased risk of MTCD HIV transmission in mothers who developed cerebral toxoplasmosis during pregnancy and they also found that congenital toxoplasmosis was associated with a 23.9 times higher risk of MTCD of HIV [30]. Further study is required to confirm whether maternal and resultant placental *T. gondii* infection significantly impacts the transmissibility of HIV to the child.

Table 1 summarises the key points regarding *T. gondii* infection in HIV-infected pregnant mothers and risks of mother MTCT. Greater efforts are needed to educate pregnant women in measures to mitigate the risk of *T. gondii* acquisition, ensure appropriate screening of HIV-infected pregnant women for *T. gondii* in pregnancy and provide timely initiation of *T. gondii* prophylaxis/treatment where indicated. In addition, novel approaches are required to achieve progress in the development of a *T. gondii* human vaccine.

**Table 1:** key points and risks (including mother-to-child transmission) of *Toxoplasma gondii* infection in HIV-infected pregnant women compared to pregnant women overall

	Pregnant women overall	HIV-infected pregnant women
*T. gondii* seroprevalence (i.e. latent infection)	33.8%	47.5%
Prevalence of acute *T. gondii* infection during pregnancy	1.1%	Unknown
Risk of MTCT of *T. gondii* from acute primary *T. gondii* infection in pregnancy	Approximately 30% overall risk (risk of MTCT of *T. gondii* is lowest in the first trimester and highest in the last trimester of pregnancy).	Unknown but likely to be partly related to the degree of immunosuppression. There is also a known additional risk in this patient group of MTCT of *T. gondii* due to reactivation.
	Risk of MTCT is reduced by up to 60% with the administration of spiramycin.	Risk reduction with the use of spiramycin is unknown-it may be similar to in the overall pregnant population but there is no specific data for spiramycin use in the HIV-infected population.
Overall risk of MTCT of *T. gondii* in pregnancy	<1%	Estimated to be <5%
Risk of disease in infant following MTCT of *T. gondii*	Risk of congenital abnormalities is highest when MTCT occurs in the first trimester and lowest in the third trimester.	It has been suggested by some that *T. gondii* infected infants born to HIV-infected mothers may have a more rapid and disseminated disease process compared to infants born to HIV-uninfected mothers, but further evidence is required to confirm this.
Diagnosis of maternal *T. gondii* infection during pregnancy and of MTCT	Toxoplasma serology is the principal method for diagnosis of maternal *T. gondii* infection. Amniocentesis (with *T. gondii* specific PCR of amniotic fluid) is required to confirm MTCT.	Depending on the degree of immunosuppression, serological markers may be less reliable in HIV-infected mothers. Additionally, *T. gondii* IgM responses may be diminished in cases reactivation/reinfection.
		Theoretical risk of HIV transmission associated with amniocentesis-this will be largely dependent on whether the mother is receiving antiretroviral therapy and is virologically suppressed.
Additional considerations	Not applicable	It is unclear whether placental inflammation due to *T. gondii* infection renders the placenta more permissive to HIV thereby increasing the risk of MTCT of HIV. It may be that this is not a significant issue in individuals who are virally suppressed but this area requires further study in order to draw any specific conclusions.

Abbreviations: *Toxoplasma gondii (T. gondii)*, mother-to-child transmission (MTCT), polymerase chain reaction (PCR)
